# Minimally invasive spine surgery strategy for congenital cervicothoracic scoliosis in children: Less blood loss and shortened segmental fusions/fewer pedical screws

**DOI:** 10.3389/fsurg.2023.1137675

**Published:** 2023-03-23

**Authors:** Zhou Zhiguo, Liu Fan, Lei Yuanxue, Wu Xing, Wang Si, Li Ruichen

**Affiliations:** ^1^ Department of Orthopedics, Wuhan Children's Hospital (Wuhan Maternal and Child Healthcare Hospital), Tongji Medical College, HUST, Wuhan, China; ^2^ Department of Rheumatology, Wuhan Children's Hospital (Wuhan Maternal and Child Healthcare Hospital), Tongji Medical College, HUST, Wuhan, China

**Keywords:** congenital scoliosis, cervicothoracic, minimally invasive spine surgery, posterior approach, hemivertebra resection

## Abstract

**Objective:**

To explore the feasibility of a minimally invasive spine surgery strategy for congenital cervicothoracic scoliosis.

**Materials and methods:**

From April 2022 to August 2022 in the hospital, three patients with torticollis and/or shoulder imbalance due to a cervicothoracic hemivertebra were performed on by hemivertebra resection and short fusion of the adjacent vertebrae. Resection was operated by a posterior approach. The average age of three patients of surgery was 8 years 2 months and the mean follow-up period was 6 months. Radiographic assessments and cosmetic outcomes were documented on changes in measurements of segmental scoliosis, neck tilt, head shift, shoulder balance, and sagittal profiles.

**Results:**

The mean operating time of the procedure was 283 min and the instrumentation density was 1.5 pedicle screws per vertebra. The mean estimated blood loss was 257 ml, which was 20% less than the data described in various literatures. The mean segmental Cobb angle at the cervicothoracic deformity was 35.9° before surgery, 20.7° after surgery, and 16.3° at the latest follow-up, with a correction rate of 54.59%. Neck tilt decreased from 17.3° before surgery to 14.3° after surgery, and 11.7° at the latest follow-up, with a correction rate of 32.37%. T1 tilt improved from 16.5° before surgery to 12.9° after surgery, and 7.6° at the latest follow-up, with a correction rate of 53.94%. The clavicle angle improved from 4.8° before surgery to 3.1° after surgery, and 1.9° at the latest follow-up, with a correction rate of 60.42%. Head shift improved from 21.4 mm before surgery to 9.2 mm after surgery, and 12.3 mm at the latest follow-up, with a correction rate of 42.52%. The correction of torticollis and shoulder asymmetry was achieved in all cases.

**Conclusions:**

Minimally invasive spine surgery strategy may be an option for congenital cervicothoracic scoliosis. A good correction of cervicothoracic dissymmetry is achieved, accompanied by fewer pedicle screws and less blood loss. By deliberate operation in young kids, surgical intervention for severe compensatory curves can be prevented.

## 1. Introduction

Congenital cervicothoracic scoliosis (CTS) poses a perplexing spinal deformity that is relatively rare and difficult to treat in young children (1–4). It mostly results from an osseous abnormality, namely hemivertebrae, block vertebrae, or junctional bar (2–5). Located at the transition zone between the relatively stiff thoracic spine and dynamic cervical segment, adjacent to the shoulders, the spinal abnormalities in this region are often associated with an obvious decompensation in the shoulders and neck, which can develop into facial asymmetry rapidly (2–6). However, due to the limited compensation in the adjacent spine segment, conservative treatment such as spine brace treatment or serial casting, has little effect (3, 5, 7). Hence, when asymmetric growth of the neck and head is proven early, surgical intervention should be recommended early in the lives of children (1, 2, 7–9).

Surgical treatment is always accompanied by dissecting and bleeding, as well as the risk of neural injury (5, 6, 10). Of the possible osseous anomalies that can result in spinal deformities, hemivertebra(HV) is one of the most common causes (1, 2, 11, 12). To date, for congenital scoliosis caused by hemivertebrae, both posterior approach alone and combined anterior and posterior approaches have been used and reported (7, 10, 13, 14). On the one hand, combined surgery provides better correction and convenience of the manipulation, but also means more incisions, blood loss, and lengthy operation time (6, 9, 15). On the other hand, for HV resection, the posterior-only procedure is less invasive by avoiding an anterior approach and has a slightly weaker correction than combined surgery (4, 9, 13, 15, 16). To our knowledge, few studies for operating in the cervicothoracic region have been reported (3–5, 8, 9, 17).

In brief, the principle of minimally invasive spine surgery (MISS) is to perform with less damage to the body and fewer complications. Given that the weight of young children with congenital CTS is often lighter than that of adults, reducing intraoperative blood loss is conducive to a safer operation (5, 11, 18). Therefore, in order to decrease surgical incisions, and minimize surgical bleeding and the fusion of segments by the instrumentations, the authors have attempted the concept of MISS for congenital cervicothoracic spine deformities *via* a posterior-only approach. The purpose of this study was to investigate the feasibility of this procedure for congenital CTS.

## 2. Materials and methods

Of the three patients in the study, two were boys and one was a girl. The patients were recruited due to torticollis and/or facial asymmetry. Their mean age at the time of surgery was 8 years 2 months (range, 6 years 3 months–10 years). The mean follow-up period was 6 months (range, 4 months−8 months). The congenital cervicothoracic scoliosis patients involved the lower thoracic region in one case and the middle and lower cervical region in one case (Table 1).

**Table 1:** Demographic anatomical and surgical characteristics.

	Age (year + month)	Gender	Resected HV	Associated congenital abnormalities	Estimated blood loss (ml)	Operating-time (min)	Fused segment	Pedicle screws
Case 1	8y + 4m	Female	C7-R	C3.T5-HV-R; C4-C6-BV; Synostosis: C2-C3-R; T4-T6-L;	450	310	2 (C6-T1)	4
Case 2	6y + 3m	Male	T2-L	C7,T1-BFV; T10 -HV-R	150	210	3 (T1-T4)	5
Case 3	10y	Male	T1-L T4-R	T2-Butterfly vertebra	170	330	5 (T1-T6)	6
Average					257	283		1.5/fusion segment

Note: HV, hemivertebra; BV, block vertebra; BFV, butterfly vertebra; R, right; L, left.

All of the patients with a cervicothoracic hemivertebra exhibited regional scoliosis and without significant kyphosis. To evaluate operative invasiveness, the volume of blood loss was reviewed from the clinical records along with transfusion and operative time. Similarly, the number of hemivertebra resection and pedicle screws was documented in our study (Table 1). The instrumentation density was also calculated using [the total pedicle screws inserted/the total instrumented vertebrae in the procedure] to assess invasiveness.

The correction ratios of both the main structural curve in the whole standing anteroposterior film and the kyphosis in the lateral standing film were evaluated. To evaluate the cosmetic parameter, the correction ratio of the T1 angle, neck tilt, and clavicle angle was investigated. For cases of head shift, a perpendicular line was drawn from the center of the mandibular, and the distance from this line to the center of the sacrum was measured to examine pre- and postoperative head balance.

Careful neurologic examination was included in the preoperative evaluation. Cervical CT angiography (CTA) and a 3-dimensional CT scan of the entire spine were performed to detect details of the vertebra and vertebral artery anomalies (3, 9, 19). A MRI was also mandatory to explore intraspinal anomalies that may also need to be addressed before surgery. Urogenital and cardiovascular examinations were performed to screen abnormalities of the renal system and congenital heart diseases.

Ethical approval was warranted by the local Ethics Committee of our institution and all the processes being performed were routine care. All subjects' guardians signed informed consent.

## 3. Radiographic assessment

Whole standing spine anteroposterior (AP) and lateral radiographs were reviewed to assess spinal correction preoperatively, postoperatively, and at the latest follow-up. The parameters in the coronal plane included both local scoliosis and the distal compensatory curve. Following Chen's method (9), four parameters were also measured to determine the cosmetic effect on each radiograph (Table 2) as follows: (1) T1 tilt, the angle between the line through the upper endplate of T1 and the horizontal line; (2) clavicle angle, the angle between the tangential line connecting the highest two points of each clavicle and the horizontal line; (3) neck tilt, the angle between the longitudinal axis of the cervical spine (the line connect the center of C7 with the center of C2 odontoid process) and the vertical line of the center of C2; and (4) head shift, the distance between the central sacral vertical line and midline of the mandibular body. For the cases of multiple hemivertebrae, the scoliosis formed by the proximal HV was defined as the proximal segmental scoliosis, compared with the curve formed by the distal HV, which was called the distal segmental scoliosis.

**Table 2:** Demographic: changes in the coronal and sagittal planes.

		T1 Tilt (°)	Clavical Angle (°)	Neck Tilt (°)	Head Shift (mm)	Segmental Scoliosis (°)
Case 1	Pre-operative	18.2	5.3	18.4	16.3	32.3
	Post-operative	14.3	4.3	10.4	10.3	16.8
	Latest follow-up	13.4	3.2	9.7	7.8	14.5
	Correction ratio %	26.37	39.63	47.53	52.15	55.11
Case 2	Pre-operative	13.6	4.5	20.5	16.3	P:34.2 D:34.6
	Post-operative	20.5	2.6	25.2	7.9	P:37.6 D:4.0
	Latest follow-up	6.5	0.5	18.7	13.3	P:10.5 D:13.4
	Correction ratio %	52.21	88.89	8.78	18.40	P:69.29 D:61.27
Case 3	Pre-operative	17.7	4.7	13.0	31.5	P:38.3 D: 39.9
	Post-operative	4.1	2.4	7.3	9.5	P:26.1 D:18.6
	Latest follow-up	3.0	2.0	6.8	15.9	P:22.5 D:20.8
	Correction ratio %	83.05	57.45	47.69	49.52	P:41.25 D:47.87

Notes: P, proximal segmental curve; D, distal segmental curve.

In the sagittal plane, segmental kyphosis, lumbar lordosis, and thoracic kyphosis were measured. The cobb angle of the segmental scoliosis curve was measured between the inferior endplate of the caudal vertebrae and the superior endplate of the cranial vertebra adjacent to the HV. The segmental lordosis or kyphosis was investigated in the sagittal plane, in the same way as was segmental scoliosis in the coronal plane. Lumbar lordosis (L1–S1) and thoracic kyphosis (T5–T12) were also assessed and documented. The distance between the posterior superior corner of the S1and C7 plumb line was obtained to assess sagittal trunk shift.

Radiographic data were assessed and collected from a picture archiving and communication system (PACS) software of our hospital, with an accuracy of 0.1°or 0.1 mm. The correction rate was calculated using [(preoperation parameter–latest follow-up parameter)/preoperation parameter] × 100%. To minimize measurement error of interobserver, all radiographs were evaluated by 2 authors who did not anticipate the surgery, and the mean measurements were collected for analysis.

## 4. Operative procedure

After general anesthesia and neuromonitoring installation, the patient was placed in the prone position with the neck slightly flexed position on the polyurethane gelatum pads of the head. The HV was checked by fluoroscopy and the back was prepared in a routine fashion. A midline skin incision was made on the back at the center of the spinal deformity. The posterior elements of the spine were carefully revealed at the level of the HV and the adjacent vertebrae. The lamina and attached transverse process of the HV was identified and pedicle screws were inserted in the adjacent vertebrae. In this study, we preferred all-pedicle-screw instrumentation based on data from the computed tomography three-dimensional (3D) reconstruction. Meanwhile, laminar hook or hybrid instrumentation should also be prepared as a good alternative. For the anomalous pedicles of the adjacent vertebrae, normal pedicle screws were shortened appropriately by a rod-cutter before insertion, consisting of a limited length of the abnormality. For the pedicles with enough diameter and length, normal pedicle screws were routinely inserted. The lamina and attached transverse process of the HV were removed to expose the pedicle after the screw implantation.

Bleeding during HV resection was well controlled by pre-cauterizing the intraspinal venous plexus down the medial wall of the pedicle to the posterior wall of the body of the HV with a bipolar coagulation. Thereafter, the pedicle was removed and the vertebral body and its discs of the HV were visualized easily. After the dura sac and nerve roots above and below the HV were carefully exposed and protected, a sharp dissection was made with a scalpel between the edge of the disc of the HV and the bony endplate of the adjacent vertebrae.Sequently, an osteotome was inserted into the gap carefully along with the bony endplate of the adjacent vertebrae. The vertebral body of the HV and the upper and lower disks were gently pried up by the osteotome and the residual anterior wall of the HV was done with a nucleus pulposus forceps and/or a curette. After the removal of the HV, a temporary rod was then placed on the concave side and the endplate of the adjacent vertebrae were completely decorticated to prepare for fusion. The temporary rod was removed and two rods were mounted on the convex and concave sides respectively. Thereafter, the gap was closed by gradually compressing the convex side and extending the concave side along the rods. Meanwhile, the upper and lower vertebrae of the HV should be horizontalized as much as possible under fluoroscopy. All the bones removed during the hemivertebrectomy were operated as graft material throughout the residual defect. Decortication of the posterolateral elements of vertebrae and fusion was then performed. Neuromonitoring was mandatory throughout the procedure.

All patients attempted to stand and walk with the drainage tube on the second day after surgery. Afterward, a cranial-cervical-thoracic brace was worn for three to six months.

## 5. Results

### 5.1. Surgical outcomes of all patients

Osteotomy and pedicle screw insertion were operated free-hand in all cases. Three young patients with congenital CTS were included in this study, and underwent posterior-only approach correction and fusion. The median operating time of the procedure was 283 min (range, 210–330 min), and the mean estimated blood loss was 257 ml (range, 150–450 ml), which was 20% less than in previous literature. The instrumentation density was 1.5 pedicle screws per vertebra, suggesting this method is less invasive. All cases achieved good shoulder balance and improved facial cosmetics.

### 5.2. Correction of the coronal plane

#### 5.2.1. Segmental correction

The mean segmental Cobb angle between the vertebrae adjacent to the HV was 35.9° before surgery, 20.7° after surgery, and 16.3° at the latest follow-up, with a correction rate of 54.59% (Table 2).

#### 5.2.2. Neck tilt

The angle between the vertical line of C2 and the longitudinal axis of the cervical spine (the line connecting the center of the C2 odontoid process with the center of C7) improved from 17.3° before surgery to 14.3° after surgery, and 11.7° at the latest follow-up, with a correction rate of 32.37%.

#### 5.2.3. T1 tilt

The angle between the line through the upper endplate of T1 and the horizontal line improved from 16.5° before surgery to 12.9° after surgery, and 7.6° at the latest follow-up, with a correction rate of 53.94%.

#### 5.2.4. Clavical tilt

The angle between the tangential line connecting the highest two points of each clavicle and the horizontal line improved from 4.8° before surgery to 3.1° after surgery, and 1.9° at the latest follow-up, with a correction rate of 60.42%.

#### 5.2.5. Head shift

The distance between a vertical line drawn from the middle line of the mandibular body to the middle of the sacrum improved from 21.4 mm before surgery to 9.2 mm after surgery, and 12.3 mm at the latest follow-up, with a correction rate of 42.52%.

### 5.3. Sagittal plane

Only subtle changes in the sagittal plane were observed. The segmental angles between the adjacent vertebrae averaged 0.9° before surgery, 1.5° after surgery, and 1.1° at the last follow-up. The mean value of LL was 32.8° before surgery, 26.5° after surgery, and 29.8° at the latest follow-up, and the mean value of TK was 25.3° before surgery, 21.3° after surgery, and 22.3° at the latest follow-up. The spinal sagittal balance was maintained perioperatively and at the final follow-up.

#### 5.3.1. Case description

**Figure 1:**
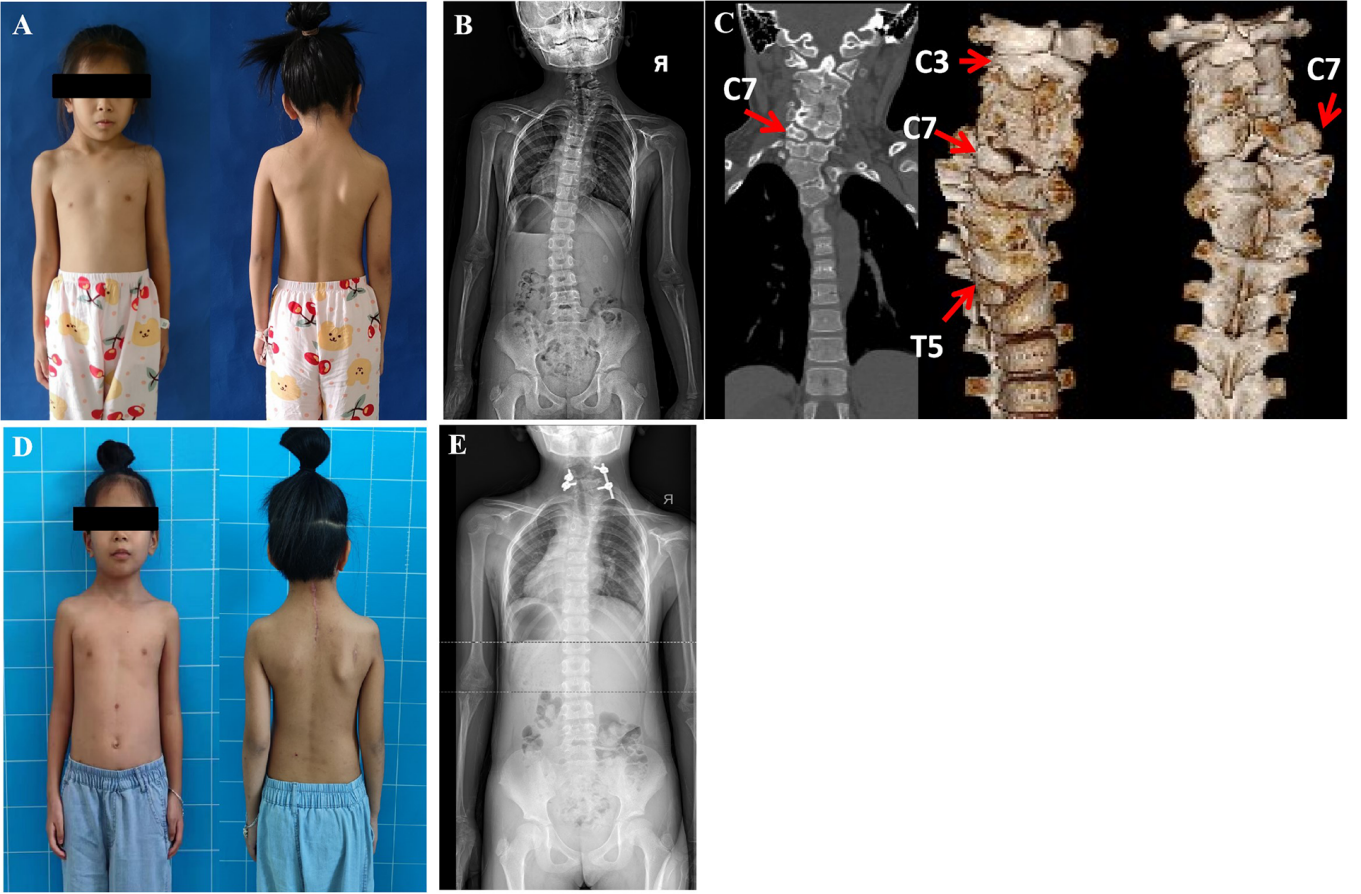
An 8 years 4 months old girl could not gaze horizontally with rigid torticollis (**A**). 3D CT showed a complexed deformitu of Klippel-Feil syndrome: C3.C7.T5-HV-R,C7 segmented HV,C3,T5 semi-segmented HV; C4-C6-BV; synostosis: C2-C3-R; T4-T6-L (**C**). She underwent C7 HVR with bilateral short fusion and could gaze horizontally three months postoperatively (**D**). Radiographs images demonstrated that there was a congenital cervicothoracic scoliosis with a compensatory lumbar curve preoperatively (**B**), thereafter, the neck tilt was significantly improved and the compensatory lumbar curve became 0° straight postoperatively at the latest follow-up 4 months later (**E**).

Case 1: A 8-year-4-month-old girl who was recognized as torticollis since the age of 6 months, and had undergone physiotherapy treatment without success. She had been unable to gaze horizontally since birth but had no pain or neurologic deficits. The preoperative computed tomography revealed a Klipple-Feil syndrome with multiple abnormalities in the cervical and upper thoracic spine: a segmented C7 HV at the right side in a synostosed bony mass from C3 to T6, two semi-segmented HV C3 and T5 at the right side, synostosed partially with the C2 to C3, and T4 to T6 conglomerate, and a blocked vertebrae C4 to C6. Meanwhile, conventional tomography showed a perplexing synostosis of the lamina of C3 to T6. The Cobb angle between C6 and T1 was 32.3°, neck tilt was 18.4° and a compensatory convex of the lumbar spine to the left was 29.3° on the standing anteroposterior x-ray (Figure 1). An MRI excluded deformities of the spinal cord. The C7 HV was removed by a posterior approach with the fusion of C6 to T1 vertebrae. C7 HV was resected with a drill and the gap between C6 and T1 was instrumented with 4 pedicle screws, with 450 ml blood loss. The dura sac and nerve roots adjacent to the C7 HV were carefully identified and protected by a retractor and the neuromonitoring did not reveal any changes intraoperatively. However, the patient underwent a transient C7 nerve injury, complaining of right shoulder pain, and inability to straighten the right upper limb and fingers, and fully recovered without treatment 3 months postoperatively. At the latest follow-up (4 months postoperative), the patient achieved horizontal gaze and her neck position was neutral (Figure 1). The Cobb angle C6 to T1 improved to 14.5° (correction ratio 55.11%) at the latest follow-up, and neck tilt was 9.7° (correction ratio 47.53%), meanwhile, her compensatory lumbar scoliosis was completely straightened.

**Figure 2:**
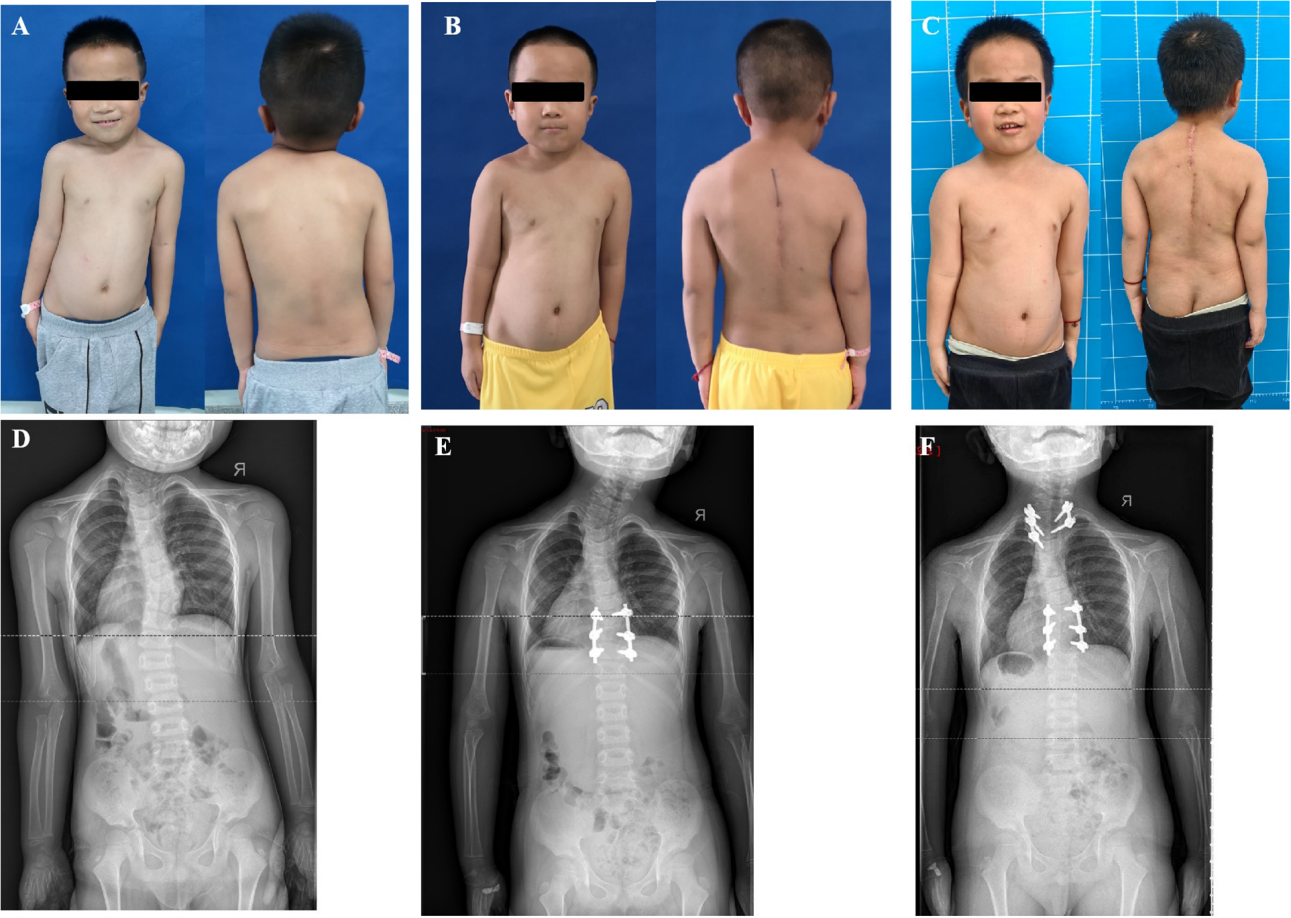
A 6 years 3 months old boy presented shoulder imbalance and facial asymmetry. Radiographs indicated that T2 and T10 were both hemivertebra and they compensated each other before the operation (**A,D**). He underwent a staged operation. The T10 HV was removed at the first surgery and he gained good shoulder balance and worsening torticollis due to the lack of compensatory T10 HV 3 months post the first operation (**B,E**). T2 HV was resected a second time, and his facial cosmesis and torticollis improved significantly 3 months post the second surgery (**C,F**).

Case 2: A 6-year-3-month-old boy presented stiff torticollis since the age of 1 year, treated by a brace without success. His torticollis worsened significantly, but there was no neck pain or neurologic deficits. Conventional tomography showed an HV between T1 and T3 on the left side and an HV between T9 and T11 on the right side of the thoracic spine. Therefore, the proximal scoliosis created by T2 HV was 34.2° and the distal thoracic curve created by T10 HV was 34.6°, compensating for each other. At the first surgery, the T10 HV was removed by a posterior approach with instrumentation T9 to T12 vertebrae, with 150 ml blood loss. Three months later, the shoulder asymmetry was corrected completely due to the correction of the distal segmental scoliosis from 34.6° to 4.0° (correction ratio 88.6%). However, without any compensation at T10 HV in the thoracic spine, a considerable neck tilt (proximal segmental scoliosis) deteriorated from 34.2°to 37.6° (Figure 2), and head shift improved from 1.62 cm to 0.79 cm. The resection of T2 HV was performed with fusion T1 to T3 vertebrae, five pedicle screws, and 150 ml blood loss. Staged HV excisions resulted in a perfect correction of neck and shoulder imbalance in general view and on radiographic assessment. At the latest follow-up (3 months post-second operation), the patient was without complaints and his neck position was neutral (Figure 2). Meanwhile, the Cobb angle at T2 HV improved from 37.6° to 10.5°, the distal curve at T10 HV from 34.6° to 13.4°, and the head shift from 0.79 cm to 1.33 cm at the last follow-up.

**Figure 3:**
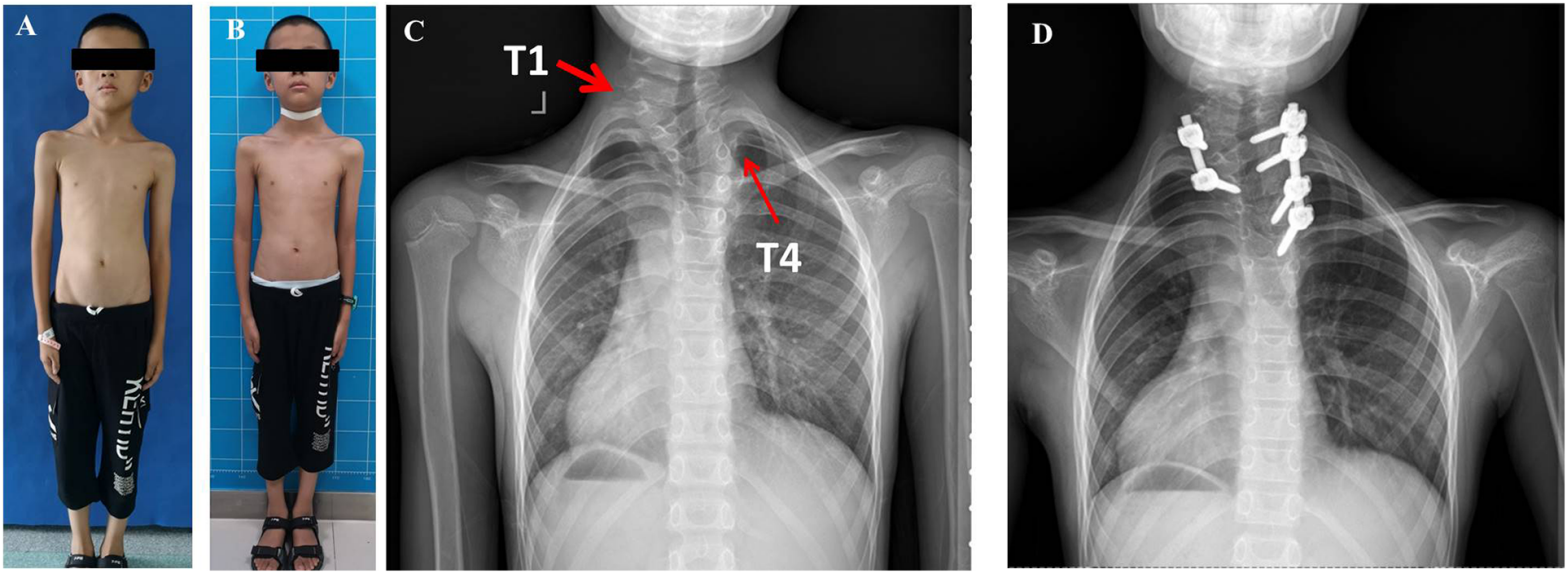
A 10 years old boy complained of neck pain and shoulder imbalance and was revealed to have two HVs at T1 and T4 (**A,C**). For the distal segment scoliosis, the T4 HV was removed and T2 to T6 vertebrae were instrumented on the convex side. For the proximal segment scoliosis, due to the difficulty of screw placement free hand in C7, a wedge osteotomy was performed between the lower part of the T1 hemivertebra and the upper part of the T2 vertebra, then the deficits were closed using screws at T1 and T3 vertebra. He achieved good shoulder balance and no neck pain 3 months postoperatively (**B,D**) Ji lilili oFIIFA.

Case 3: A 10-year-old boy complained of neck pain with mild torticollis and right shoulder height since the age of 9 years, without physiotherapy treatment. The radiologic examinations revealed a right clavicle height with an HV between C7 and T2 vertebrae on the left side and an HV between T3 and T5 vertebrae on the right side. T4 HV was removed first and the initial plan for T1 HV was resection of T1 HV and short fusion between C7 and T2 vertebrae. Due to the difficulty of screw insertion free hand in the C7 vertebra, our strategy was adjusted to preserve the pedicle and upper part of T1. A wedge osteotomy was performed between the lower part of the T1 HV and the upper part of the T2 vertebra, then the deficits were closed using screws at T1 HV and T3 vertebra. After the osteotomy at T4 and partial T1 HV, six pedicle screws were done to fix five spinal segments (T1-T6) and 170 ml of bleeding was documented. At the latest follow-up, the patient was pain-free and his shoulder balance was restored effectively. The neck tilt was 6.8° to the right and the head shift 1.59 cm to the left at the latest follow-up (Figure 3).

### 5.3.2. Complications

Although there was no abnormal change from the neuromonitoring intraoperatively, case 1 presented a transient C7 nerve injury, complaining of right shoulder pain, inability to straighten the right upper limb and fingers, and fully recovered 3 months without treatment after surgery. No surgical site infection occurred in all cases.

## 6. Discussion

The average amount of blood loss in this study was 257 ml, about 20% less than the median blood loss of 313 ml in previous literatures (3, 5, 7, 9, 16, 17). In this study, 1.5 pedicle screws were instrumented per vertebral body, a reduction of 0.5 screws per vertebral body compared to the usual 2 pedicle screws per vertebral body (3, 16, 17). For CTS, the fusion length could be reduced by early intervention and the pedicle screws could be cut down by appropriate screw density, which was to reduce body damage. For multiple hemivertebra of CTS, priority treatment to the distal hemivertebra and staged surgery is an appropriate option to employ the principle of MISS strategy. Following the above measures, the fixed segment could be reduced and the amount of bleeding could be controlled efficiently.

Whereas there were relatively few cases of congenital scoliosis in the cervicothoracic segment, current surgical treatments vary widely in previous literature. Current posterior-only surgery and combined surgery have their own pros and cons, respectively (13–21). Therefore, to provide satisfactory scoliosis correction and less damage to the body of children, surgical timing, surgical plan-making, and surgical techniques are important foundations for MISS strategy.

First, surgical timing is a priority principle of MISS to treat congenital CTS. Timing the operation properly means shorter fusion segments and fewer pedicle screws. Case 1 presented a perplexing cervicothoracic spinal deformity and compensatory lumbar scoliosis on radiographic imaging (Figure 1). After excision of the C7 HV that was the apex, a simplified bifusion of the upper and lower vertebrae was performed. The patient's torticollis was efficiently improved with the compensatory lumbar curve spontaneously corrected to 0° (Figure 1). This result suggested that HV excision at an early age may arrest the secondary curve progression of trunk shift. A similar conclusion was obtained that early operation to congenital CTS could meliorate overall spine coronal balance significantly after surgery (2, 5, 9, 10, 15). Conversely, the untreated compensatory curves tend to progress to structural deformity, which requires extended correction and fusions (2, 7, 17). Hence, in the cases of congenital scoliosis at the cervicothoracic region in children, shoulder imbalance and cosmetic deficit rather than the angle of curvature is a critical indications for operative treatment (2, 3, 5, 15–17).

Second, for multiple hemivertebrae in congenital CTS, it was an optimal option that the flexible distal HV segment should be treated first, sequentially followed by the proximal HV region with poor mobility. This surgical procedure could indicate neck and shoulder balance as good as possible with fewer fusions (9, 20). Case 2 was treated with staged hemivertebrae resection (Figure 2). The thoracic HV at T10 was resected at the first time, and the shoulder balance was significantly improved while the deterioration of his torticollis was noted at the follow-up post first surgery. The second surgery was performed to remove the cervicothoracic HV at T1 three months after the first operation, and the torticollis was significantly improved both immediately after surgery and at the latest follow-up. In case 3, the two hemivertebrae at T1 and T4 were adjacent to each other, and a one-stage surgery was performed. The caudal HV at T4 was firstly removed and instrumented first. Consequently, a wedge osteotomy was performed between T1 HV and T2 vertebra, then, the gap was closed using internal fixation at T1 HV and T3 vertebra a one-stage operation. Thereafter, four pedicle screws were used to fix the distal convex side and two screws instrumented the proximal convex side (Figure 3). The purpose of this asymmetrical screw formation was to warrant a solid stabilization of the pedicle screws on the convex side and reduce the number of instrumentations (21). Eventually, both patients achieved good shoulder balance and facial cosmetics. Therefore, for a complex congenital CTS, more attention should be paid to reasonable surgical plan-making (13, 19–21).

Third, the surgical technique of meticulous pre-cautery of the intraspinal venous plexus adjacent to the hemivertebrae can effectively decrease the amount of intraoperative bleeding, consistent with the MISS strategy. After the intraspinal venous plexus pre-hemostasis was completed, there was little bleeding in the surgical field during HV resection. Moreover, in case of massive bleeding during the resection of the hemivertebra, effective hemostasis could be achieved by cauterization of the venous plexus down the posterior wall of the vertebral body. The average amount of blood loss was about 257 ml in our study, which was 20% less than the procedures as being described in the previous literatures. For young children, lower operation time and blood loss can ensure safer operations and faster recovery (5, 19, 21). Hence, the effective maneuver of intraspinal pre-hemostasis is more conducive to the practice of MISS strategy (19, 21).

There were several limitations in our study. First, it had a limited sample size since this circumstance is relatively rare. However, to the best of our knowledge, this is an earlier study to treat congenital CTS with MISS strategy. The second limitation of this study is that the follow-up duration is still relatively short considering the immature nature of the spines in question. Thus, a long-term follow-up study should be further carried out.

In conclusion, our short-term study achieved excellent correction results using the one-stage strategy with MISS, which hopefully could provide an option for the treatment of congenital CTS. Future well-designed prospective studies with longer follow-up times are required to further validate our study.

## Data Availability

The original contributions presented in the study are included in the article/Supplementary Material, further inquiries can be directed to the corresponding author.
